# The Quantum Tunneling of Ions Model Can Explain the Pathophysiology of Tinnitus

**DOI:** 10.3390/brainsci12040426

**Published:** 2022-03-23

**Authors:** Baeth M Al-Rawashdeh, Abdallah Barjas Qaswal, Aiman Suleiman, Fuad Mohammed Zayed, S. M. Al-Rawashdeh, Mohamed Tawalbeh, Lubna Khreesha, Ayham Alzubaidi, Enas Al-Zubidi, Zuhir Ghala, Ahmad Almasri, Mohammed Yasein, Khaled Ojjoh, Ahmad Alraiqib, Mohammad Iswaid, Murad Emar, Shahed Haimour, Ala’ Saifan, Zaid Mahameed

**Affiliations:** 1 Department of Special Surgery, Jordan University Hospital, School of Medicine, The University of Jordan, Amman 11942, Jordan; tawalbeh@ju.edu.jo (M.T.); l.khreesha@ju.edu.jo (L.K.); 2 School of Medicine, The University of Jordan, Amman 11942, Jordan; fuad.41994@gmail.com (F.M.Z.); khaledojjoh@gmail.com (K.O.); mohd_iswaid@hotmail.com (M.I.); 3 Department of Anesthesia, Intensive Care and Pain Management, Beth Israel Deaconess Medical Center, Harvard Medical School, Boston, MA 02215, USA; asuleima@bidmc.harvard.edu; 4 Department of Scientific Basic Sciences, Faculty of Engineering Technology, Al-Balqa Applied University, Salt 19117, Jordan; sokainah.rawashdeh@bau.edu.jo; 5 Department of Internship Program, Jordan University Hospital, Amman 11942, Jordan; ayhamaalzubidi@gmail.com (A.A.); enasoalz12@gmail.com (E.A.-Z.); zohair.ghalla@gmail.com (Z.G.); ahmadosama98@hotmail.com (A.A.); mohammedessa98@gmail.com (M.Y.); shahed_haymoor@hotmail.com (S.H.); ahss1997@gmail.com (A.S.); zaid.m.mahameed@gmail.com (Z.M.); 6 School of Medicine, Mutah University, Karak 61710, Jordan; ahmadfayez813@yahoo.com; 7 Department of General Surgery, Leicester University Hospitals, P.O. Box 7853, Leicester LE1 9WW, UK; morad.aamar@yahoo.com

**Keywords:** tinnitus, quantum tunneling, quantum biology, inner hair cell, quantum conductance, voltage-gated channel, potassium ion, sodium ion, calcium ion

## Abstract

Tinnitus is a well-known pathological entity in clinical practice. However, the pathophysiological mechanisms behind tinnitus seem to be elusive and cannot provide a comprehensive understanding of its pathogenesis and clinical manifestations. Hence, in the present study, we explore the mathematical model of ions’ quantum tunneling to propose an original pathophysiological mechanism for the sensation of tinnitus. The present model focuses on two major aspects: The first aspect is the ability of ions, including sodium, potassium, and calcium, to depolarize the membrane potential of inner hair cells and the neurons of the auditory pathway. This membrane depolarization is induced via the quantum tunneling of ions through closed voltage-gated channels. The state of membrane depolarization can be a state of hyper-excitability or hypo-excitability, depending on the degree of depolarization. Both of these states aid in understanding the pathophysiology of tinnitus. The second aspect is the quantum tunneling signals between the demyelinated neurons of the auditory pathway. These signals are mediated via the quantum tunneling of potassium ions, which exit to the extracellular fluid during an action potential event. These quantum signals can be viewed as a “quantum synapse” between neurons. The formation of quantum synapses results in hyper-excitability among the demyelinated neurons of the auditory pathway. Both of these aspects augment and amplify the electrical signals in the auditory pathway and result in a loss of the spatiotemporal fidelity of sound signals going to the brain centers. The brain interprets this hyper-excitability and loss of spatiotemporal fidelity as tinnitus. Herein, we show mathematically that the quantum tunneling of ions can depolarize the membrane potential of the inner hair cells and neurons of the auditory pathway. Moreover, we calculate the probability of action potential induction in the neurons of the auditory pathway generated by the quantum tunneling signals of potassium ions.

## 1. Introduction

Tinnitus is defined as the conscious perception of a phantom sound or noise in the ear or in the head [1]. It is frequently linked to noise-induced hearing loss and presbycusis [1,2]. Risk factors that increase the likelihood of getting tinnitus include vascular injury, hypertension, diabetes, autoimmune disorders, head injury, and degenerative neural disorders [1,2,3].

However, the exact pathophysiological mechanisms behind the generation and maintenance of tinnitus are not clearly defined [1,2,3]. It is thought that tinnitus is generated and maintained due to aberrant neuronal activity that can be located at any site along the auditory pathway from the cochlea to the auditory brain centers [1,4]. Accordingly, tinnitus can be classified into three main categories: (1) cochlear tinnitus, (2) peripheral-dependent central tinnitus, and (3) peripheral-independent central tinnitus [1,5]. Cochlear tinnitus refers to the altered neuronal activity in the cochlea, which is transmitted through the auditory pathway to the hearing brain centers and is perceived as tinnitus. Peripheral-dependent central tinnitus refers to the perception of tinnitus in brain centers due to higher signal inputs from the peripheral auditory pathway, while peripheral-independent central tinnitus refers to the perception of tinnitus in brain centers independent from the sound signals coming from the periphery [1,5].

Furthermore, the higher frequency of sound signals traveling from the periphery to the brain centers has been linked to depolarization in the membrane of cochlear inner hair cells [1,6,7]. As the membrane potential of inner hair cells becomes depolarized, the spontaneous activity of the cochlea increases; thus, higher electrical inputs enter the hearing centers in the brain, which perceives this as tinnitus [1,6,7]. On the other hand, other pathological entities may decrease spontaneous cochlear activity, resulting in neuronal compensation in the brain, which is also perceived as tinnitus due to aberrant neuronal activity in the central auditory circuits [1,8]. The decreased activity of the cochlea is observed in hearing loss, which explains its strong association with tinnitus [1]. Accordingly, two major aspects are implicated in the pathophysiology of tinnitus: (1) the depolarization of the membrane potential of inner hair cells, which increases cochlear spontaneous activity, and (2) abnormal neuronal activity in the peripheral and central auditory pathways.

However, the mechanisms of inner hair cell depolarization are poorly defined and cannot provide consistent explanations. For example, it has been proposed that the closure of mechano-electrical transduction (MET) channels results in an increase in the endocochlear potential (endolymphatic potential), which in turn depolarizes the inner hair cells [1,9]. This mechanism seems to contradict the normal physiological action of MET channels because the opening of these channels results in the flow of potassium ions into the cell, which leads to depolarization [10]. Consistently, it has been found that the loss of tall stereocilia at the apical membrane of inner hair cells leads to a decrease in the inward cationic currents, which induces hyperpolarization in the membrane of inner hair cells instead of depolarization [11]. This hyperpolarization decreases the spontaneous activity of the cochlea [11]. Hence, it is the opening of MET channels, not the closure, that leads to depolarization [10]. Other proposed mechanisms focus on the outer hair cells and their discordance with inner hair cells [12]. Such discordance states that the early damage of outer hair cells before inner hair cells causes the tectorial membrane to impinge on the stereocilia of inner hair cells, and thus a depolarization in their membrane occurs [12]. The discordance mechanism may explain the depolarization in the early phases of tinnitus, but not in the chronic phases of tinnitus in which both the inner hair cells and outer hair cells are damaged. In addition, such an impingement does not necessarily guarantee the opening of MET channels at the apical stereocilial membrane because the mechanical force of the impingement may result in hyperpolarization or depolarization according to its direction, which is the same principle applied to the direction of stereocilial deflection [12]. Furthermore, it is unclear how tinnitus risk factors, such as noise and aging, can result in abnormal neuronal activity in the peripheral and central auditory pathways [1,12]. In addition, there is no conclusive pathophysiological basis to explain how such abnormal activity causes tinnitus and how it can affect the hearing process [1,12]. These challenges will be addressed and conceptualized in the context of the quantum tunneling model.

In the present study, we will try to explain the mechanisms behind the membrane depolarization of inner hair cells and the aberrant neural activity by exploiting the model of ions’ quantum tunneling through voltage-gated channels. This model will be rationally utilized in the context of risk factors for tinnitus. The mathematical model of *ion* quantum tunneling proposes the ability of ions, such as sodium and potassium ions, to tunnel through the closed gates of channels [13,14,15]. This model has been used before to explain several biological and pathological processes and actions [16,17,18], particularly phantom limb pain [19], which is commonly used as an analogy to the pathophysiology of tinnitus [20].

The general approach of the present study will focus on two main ideas: (1) We will show that the quantum tunneling of ions, including sodium, potassium, and calcium, through the closed gates of *ion* channels can directly depolarize the membrane potential of inner hair cells and the neurons of the auditory pathway. This membrane depolarization is considered to enhance cochlear firing rates. (2) We will show that when the neurons of the auditory pathways become demyelinated due to noise trauma and presbycusis [12,21], they exhibit a “quantum synapse” or “quantum crosstalk” between neurons. This quantum communication between neurons allows for an action potential to be induced in the neighboring neurons, which results in hyper-excitability and aberrant neuronal activity in the peripheral or central pathways. Accordingly, we aim to show that the quantum tunneling model has the potential to provide reasonable explanations for inner hair cell depolarization and aberrant neuronal activity, and their relation to tinnitus. These features will be elaborated further in Section 2, Section 3 and Section 4.

## 2. Mathematical Model of the Quantum Tunneling of Ions

### 2.1. Mathematical Equations That Describe the Quantum Tunneling-Induced Membrane Depolarization of Inner Hair Cells

**Figure 1:**
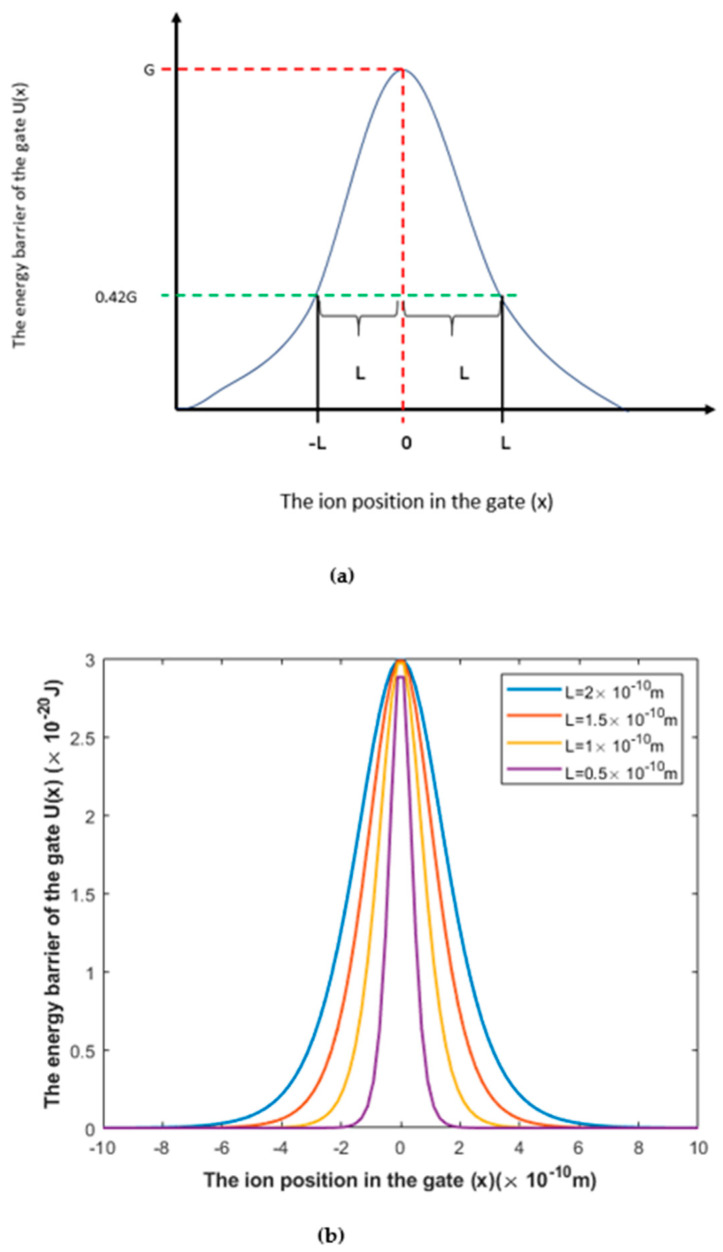
The symmetric Eckart potential. (**a**) Schematic diagram of the symmetric Eckart potential in which *G* is the barrier height and *L* is the gate length at which *U*(*L*) = 0.42 *G*, according to Equation (1). (**b**) Actual plots of the symmetric Eckart potential at different gate lengths *L*.

A model of the quantum tunneling of ions through closed gates has been proposed [13,14,15] and used to explain several physiological, pathological, and pharmacological actions [16,17,18]. The major focus of the quantum model is on voltage-gated channels [13,14,15]. These channels seal off the permeation of ions by forming a narrow hydrophobic constriction on the intracellular side, which represents the closed gate [22,23,24,25]. However, the location of the closed gate may change according to the state of the channel (resting state or inactivated state) [26,27,28,29]. The quantum model views the closed gate as an energy barrier with a height sufficient to block the kinetic permeation of ions [22,23,24,25]. It states that the ions have a non-zero probability of passing through the closed gate, even though the energy height of the gate is higher than the kinetic energy of the *ion* [13,14,15]. In previous studies that focused on the tunneling probability of ions [13,14,15], a triangular barrier shape of the closed gate was used to approximate the barrier shape obtained experimentally using potential mean force (PMF) calculations [30,31,32,33]. However, in our study, we use the symmetric Eckart potential [34,35,36] to approximate the barrier shape because it gives a better estimation of tunneling probability (see Figure 1). The Eckart potential can better mimic and approximate the experimentally obtained barrier shape [30,31,32,33,37,38,39] than the previously used triangle shape can because it takes into account the non-linearity of the barrier shape [30,31,32,33,37,38,39] in a better fashion than the triangle shape. We use approximate barrier shapes because there is no definitive mathematical equation that describes the potential energy barrier of ions with respect to the ion’s position in the gate; thus, we depend on the experimentally obtained curves to carry out the mathematical calculations.

The symmetric Eckart potential can be mathematically represented by the following equation [34,35,36]:

(1) $$U(x)=\frac{G}{\cosh^{2}(\frac{x}{L})},$$

where *U*(*x*) is the energy barrier of the gate, *G* is the barrier height of the gate, *x* is the *ion* position in the gate, and *L* is the barrier width at which U(x)=0.42G. We will use the term “the length of the gate” for *L* throughout the paper.

A schematic diagram and actual plots for the symmetric Eckart potential are represented in Figure 1.

If the tunneling probability of a particle through the Eckart potential is considered, the tunneling probability can be calculated by the following equation [34,35,36]:

(2) $$T_{Q}=\frac{\cosh(2\pi (2\alpha ))-1}{\cosh(2\pi (2\alpha ))+\cosh(2\pi \delta )},$$

where $\alpha =\frac{L}{2\hbar }\sqrt{2mE_{K}}$, $\delta =\frac{1}{2}\sqrt{(\frac{16\pi^{2}L^{2}}{h^{2}})2Gm-1}$ (the −1 under the square root will be neglected in the following calculations because it does not significantly affect the results), *L* is the length of the gate (at which *U*(*L*) = 0.42 *G*), *m* is the mass of the ion, $E_{K}$ is the kinetic energy of the ion, *G* is the barrier height of the gate, *h* is the Planck constant ($6.6\times 10^{-34}$ Js), and ℏ is the reduced Planck constant ($1.05\times 10^{-34}$ Js).

To reduce the complexity of the mathematics in Equation (2) while maintaining the consistency and reasonability of the numerical results, the following approximation can be used [34]: $\cosh x\approx \frac{1}{2}e^{x}$ for any x≥3. This can be easily checked by substituting the following values: $L=0.5\times 10^{-10}$ m, $m_{Na}=3.8\times 10^{-26}$ Kg, $E_{K}=1\times 10^{-20}$ J, and $G=1\times 10^{-20}$ J in α and δ. These values will become clear in the following sections.

Accordingly, Equation (2) can be re-written as the following [34]:

(3) $$T_{Q}\approx \frac{e^{2\pi .2\alpha }}{e^{2\pi .2\alpha }+e^{2\pi .\delta }}\;\approx \frac{1}{\frac{e^{2\pi .2\alpha }+e^{2\pi .\delta }}{e^{2\pi .2\alpha }}}\;\approx \frac{1}{1+e^{2\pi (\delta -2\alpha )}}\approx e^{-2\pi (\delta -2\alpha )},$$

The −1 and 1 are neglected in Equations (2) and (3), respectively, because they do not significantly affect the results.

Eventually, by substituting the mathematical expressions of α and δ in Equation (3), we find that the tunneling probability through a symmetric Eckart potential can be calculated by the following equation:

(4) $$T_{Q}=e^{\frac{-\sqrt{8\pi^{2}m}}{\hbar }L(\sqrt{G}-\sqrt{E_{K}})},$$

where $T_{Q}$ is the quantum tunneling probability of the ion, *m* the mass of the ion, ℏ is the reduced Planck constant, *L* is the length of the gate, *G* is the barrier height of the gate, and $E_{K}$ is the kinetic energy of the ion.

The ions are mainly present in two compartments: (1) inside the inner hair cells (the intracellular ions) and (2) in the perilymph (the extracellular ions). Moreover, the closed activation gate in the voltage-gated channels is located at the intracellular end of the cellular membrane [22,23,24,25]. When the extracellular ions enter the channel, they gain kinetic energy as they pass through the membrane potential of the inner hair cell, which is negative on the inside relative to the outside. In addition, thermal kinetic energy will be added to the total kinetic energy [40,41] once they hit the closed intracellular gate. On the other hand, the intracellular ions will benefit only from the thermal kinetic energy once they reach the closed intracellular gate, since they hit the intracellular gate before going through the membrane potential.

Accordingly, the respective total kinetic energies of the extracellular and intracellular ions are:

(5) $$E_{K(E)}=qV_{m}+\frac{3}{2}K_{B}T,$$

(6) $$E_{K(I)}=\frac{3}{2}K_{B}T,$$

where $K_{B}$ is the Boltzmann constant ($1.38\times 10^{-23}$ J/K), *T* is body temperature (310 K), *q* is the charge of the ion, and *V_m_* is the membrane potential.

**Figure 2:**
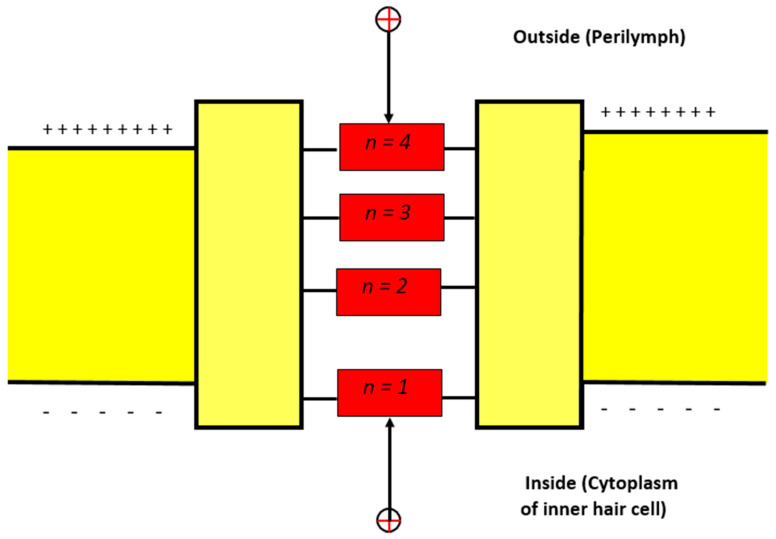
Schematic diagram for the possible locations of the closed activation and inactivation gates.

The model of quantum tunneling can be applied to other closed states in which the inactivation gate is formed. The inactivation gate can be located at the intracellular end or somewhere between the intracellular and extracellular ends [26,27,28,29]. To account for the gate location and its effect on the tunneling probability, we will assign a number from 1–4 to account for the location of the gate (Figure 2). The purpose of this numbering is to determine the membrane potential available for the extracellular ions to add on to their kinetic energy according to the equation $\frac{V_{m}}{n}$. For example, if *n* = 2, then the membrane potential available for addition is $\frac{V_{m}}{2}$.

Therefore, the respective tunneling probabilities of extracellular and intracellular ions are:

(7) $$T_{Q(E)}=e^{\frac{-\sqrt{8\pi^{2}m}}{\hbar }L(\sqrt{G}-\sqrt{q\frac{V_{m}}{n}+\frac{3}{2}K_{B}T})},$$

(8) $$T_{Q(I)}=e^{\frac{-\sqrt{8\pi^{2}m}}{\hbar }L(\sqrt{G}-\sqrt{\frac{3}{2}K_{B}T})},$$

As a result, the quantum unitary conductance of a single channel can be calculated by the following equation [42,43]:

(9) $$C_{Q}=\frac{q^{2}}{h}T_{Q},$$

where $C_{Q}$ is the quantum unitary conductance of the closed channel in Siemens (S), *q* is the charge of the *ion* ($q_{Na,K}=1.6\times 10^{-19}$ C and $q_{Ca}=3.2\times 10^{-19}$ C), *h* is the Planck constant, and $T_{Q}$ is the tunneling probability of the ions. For monovalent ions, such as sodium and potassium ions, $\frac{q^{2}}{h}=3.88\times 10^{-2}$ mS, while for divalent ions $\frac{q^{2}}{h}=15.52\times 10^{-2}$ mS.

Hence, the quantum membrane conductance can be calculated by the following equation [44,45]:

(10) $$MC_{Q}=C_{Q}\times D,$$

where $MC_{Q}$ is the quantum membrane conductance, $C_{Q}$ is the quantum unitary conductance of the closed channel, and *D* is the channel density (channels/cm^2^). The unit for quantum membrane conductance used in the present study is mS/cm^2^.

**Figure 3:**
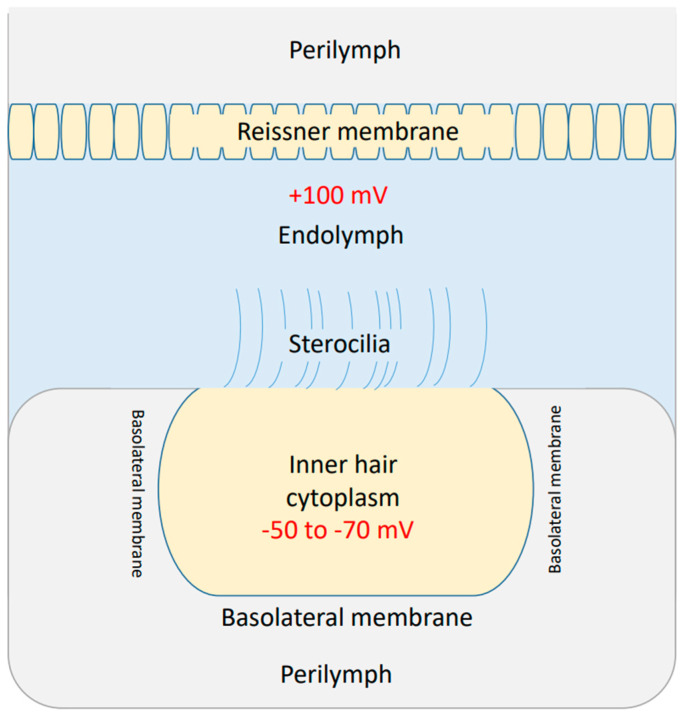
The three major compartments in the cochlea: the perilymph, the endolymph, and the cytoplasm of the inner hair cell. The endolymphatic potential is around + 100 mV and the potential inside the inner hair cell is around −50 to −70 mV.

There are three membranes in the cochlea through which the flow of ions can affect the cochlear potential. These three membranes are: (1) Reissner’s membrane, which separates the perilymph from the endolymph [46]. (2) The apical membrane with stereocilia, which separates the endolymph from the cytoplasm of inner hair cells [46]. (3) The basolateral membrane [46], which separates the cytoplasm of inner hair cells from the perilymph. See Figure 3.

The endolymphatic (endocochlear) potential is around +100 mV, which is positive inside the endolymph, and the perilymph is considered to be neutral [46]. This positive potential emerges as a result of sodium flow through Reissner’s membrane to the endolymph [46]. The efflux of potassium ions through the basolateral membrane of inner hair cells to the perilymph results in a membrane potential of −50 to −70 mV, which is negative inside the inner hair cell with respect to the perilymph [46]. Moreover, the deflection of stereocilia at the apical membrane results in a change in its permeability to potassium ions, which can depolarize or hyperpolarize the inner hair cells [46]. The difference between the endolymphatic potential of +100 mV and the potential inside the inner hair cells (−50 to −70 mV) is called the cochlear potential [46], and is around 150 mV to 170 mV. The cochlear potential represents a major driving force for potassium ions to flow through the apical stereocilial membrane to depolarize the inner hair cells and transmit sound waves as electrical signals to the auditory neuronal pathway.

We will focus on the changes in the membrane potential at the basolateral membrane of the inner hair cells themselves, which contributes around 50–70 mV of the overall cochlear membrane potential. The decrease in the membrane potential at the basolateral membrane (depolarization) leads to a net depolarization of the cochlear potential.

Applying the classical version of the Goldman–Hodgkin–Katz (GHK) equation [44,45,46] to the basolateral membrane of inner hair cell results in the following equation:

(11) $$MC_{Na}{\left[Na\right]}_{E}+MC_{K}{\left[K\right]}_{E}=e^{\frac{-FV_{m}}{RT}}(MC_{Na}{\left[Na\right]}_{I}+MC_{K}{\left[K\right]}_{I}),$$

where ${\left[K\right]}_{E}=5$ mmol/L [46] is the potassium concentration in the perilymph outside the inner hair cell, ${\left[Na\right]}_{E}=140$ mmol/L [46] is the sodium concentration outside the inner hair cell (in perilymph), ${\left[K\right]}_{I}=120$ mmol/L [46] is the potassium concentration inside the inner hair cell, ${\left[Na\right]}_{I}=15$ mmol/L [46] is the sodium concentration inside the inner hair cell, $MC_{K}=0.5$ mS/cm^2^ [44,45,46] is the leak membrane conductance of potassium ions at the resting state, and $MC_{Na}=0.01$ mS/cm^2^ [44,45,46] is the leak membrane conductance of sodium ions at the resting state. The ratio between $MC_{K}$ and $MC_{Na}$ is 100 to 2, which is the same as the ratio reported in [46], $V_{m}$ is the resting membrane potential of the inner hair cell, *F* is Faraday’s constant (96,485.33 C/mol), *R* is the gas constant (8.31 J/Kmol), and *T* is absolute body temperature (310 K). The minus sign is added to the term $e^{\frac{-FV_{m}}{RT}}$ to obtain an absolute value of the membrane potential, which is negative inside with regard to the outside. Throughout the paper, the value of the membrane potential is referred to as an absolute value (positive). When the above parameters are substituted in Equation (11), $V_{m}=0.073$ V, which is near the normal membrane potential of inner hair cells at the basolateral sides (50 to 70 mV) [46].

To integrate quantum conductance into this model, the quantum version of the GHK equation for monovalent ions, such as sodium and potassium ions [14,15], should be used as follows:

(12) $$MC_{Na}{\left[Na\right]}_{E}+MC_{K}{\left[K\right]}_{E}+MC_{Q-ion(E)}{\left[ion\right]}_{E}=e^{\frac{-FV_{m}}{RT}}(MC_{Na}{\left[Na\right]}_{I}+MC_{K}{\left[K\right]}_{I}+MC_{Q-ion(I)}{\left[ion\right]}_{I}),$$

where $MC_{Q-ion(E)}$ is the quantum membrane conductance of extracellular ions (in the perilymph), $MC_{Q-ion(I)}$ is the quantum membrane conductance of intracellular ions (inside the inner hair cells), ${\left[ion\right]}_{E}$ is the concentration of the *ion* in the perilymph, and ${\left[ion\right]}_{I}$ is the concentration of the *ion* inside the inner hair cells. The *ion* in Equation (12) can be a sodium *ion* or potassium ion.

However, when divalent ions, such as calcium ions, are considered, the following equation must be applied:

(13) $$(S1-S2)+\sqrt{{\left(S1+S2\right)}^{2}+4(S1H2+S2H1+H1H2)}=2e^{\frac{-FV_{m}}{RT}}(S2+H2),$$

where $S1=MC_{Na}{\left[Na\right]}_{E}+MC_{K}{\left[K\right]}_{E}$, $S2=MC_{Na}{\left[Na\right]}_{I}+MC_{K}{\left[K\right]}_{I}$, $H1=MC_{Q}{\left(Ca\right)}_{E}{\left[Ca\right]}_{E}$, $H2=MC_{Q}{\left(Ca\right)}_{I}{\left[Ca\right]}_{I}$.

Equation (13) has been used and applied to divalent magnesium ions, and the full derivation can be found in [47].

By exploiting these equations, we showed how the quantum tunneling of ions can change the membrane potential of an inner hair cell from 0.073 V to lower potential values to delineate the depolarization effect mediated by the quantum membrane conductance.

### 2.2. Mathematical Equations That Describe the Probability of Inducing an Action Potential in Demyelinated Neurons of the Auditory Pathway (the Formation of a Quantum Synapse)

**Figure 4:**
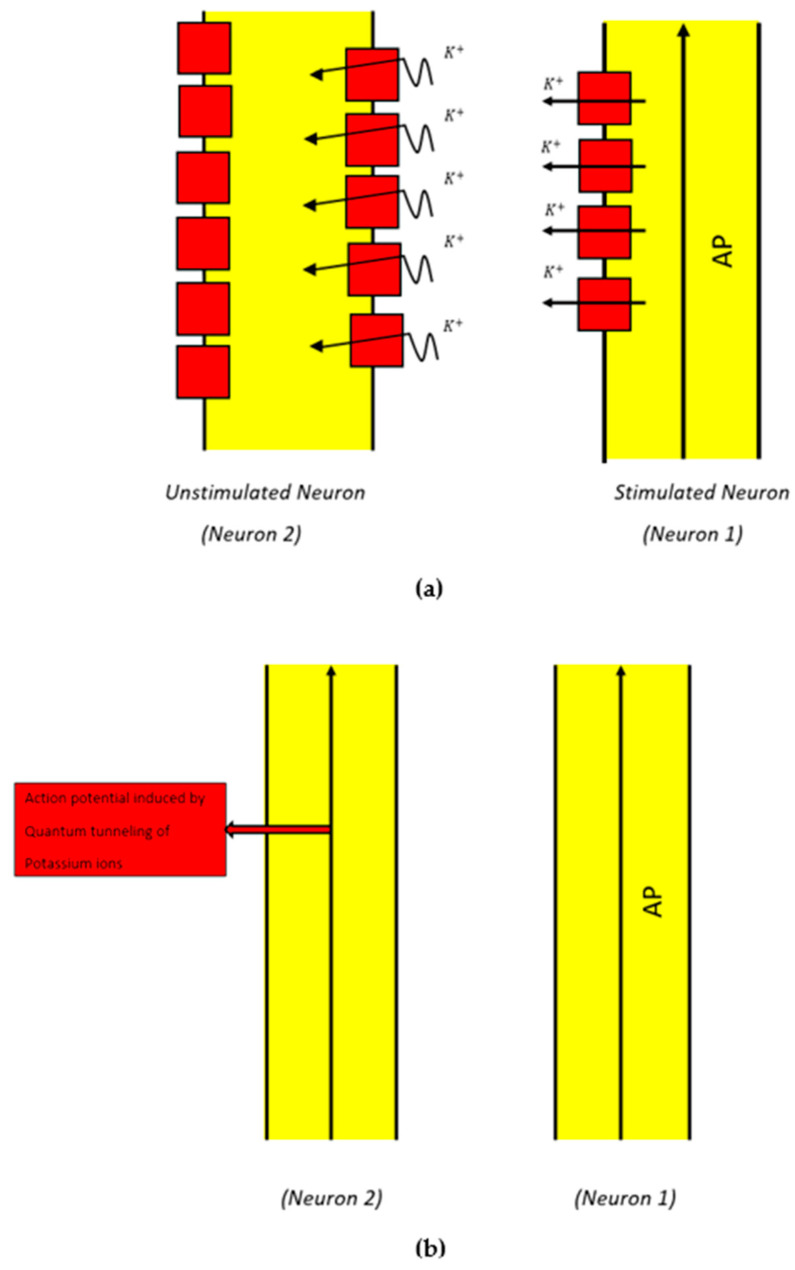
Schematic diagram of the quantum synapse between two neurons. (**a**) Neuron 1 carries an action potential (AP). During action potential propagation, potassium ions exit to the outside when potassium channels (represented in red ) open. The classical passage of ions through open channels is indicated by the straight arrows. These potassium ions also have the chance to tunnel through the closed exposed potassium channels in the membrane of an adjacent unstimulated neuron (Neuron 2) that has been demyelinated. The quantum tunneling of potassium ions through the closed channels is indicated by the wavy arrows. (**b**) An action potential is induced in Neuron 2 due to the depolarization mediated by the quantum tunneling of potassium ions.

The basic idea behind the formation of a “quantum synapse” between the demyelinated neurons of the auditory pathway is that when an action potential is propagated through a neuron, there will be a probability that this stimulated neuron will induce an action potential in an adjacent unstimulated neuron. This action potential induction is achieved via the quantum tunneling of potassium ions through the closed potassium channels, which are exposed upon demyelination after being covered by the myelin sheath [48,49,50,51] (Figure 4).

During action potential generation, there will be an increase in the extracellular potassium concentration [52]. We assume that there are $1.37\times 10^{6}$ potassium ions per $314\;\mu m^{2}$ of the neuronal membrane ($4.36\times 10^{3}$ ions/$\mu m^{2}$) [16,45], which exit during an action potential. Moreover, a neuron with a length L=100 μm and axonal radius r=0.5 μm can result in a surface area of $314\;\mu m^{2}$ and an intracellular neuronal volume of $78.5\;\mu m^{3}$ (assuming that the neuron takes the shape of a cylinder). Accordingly, the extracellular volume that potassium ions diffuse into can be estimated as $52.6\;\mu m^{3}$ (assuming the ratio between the extracellular and intracellular volumes is 0.67 [45,53]).

Accordingly, the increase in the extracellular potassium concentration can be calculated by the following equation:

(14) $${\left[K\right]}_{AP}=\frac{N_{AP}}{N_{A}V_{E}},$$

where ${\left[K\right]}_{AP}$ is the magnitude of the increase in the extracellular potassium concentration during the action potential, $N_{AP}$ is the number of potassium ions that exit to the extracellular compartment per unit surface area and per an action potential, $N_{A}$ is Avogadro’s number, and $V_{E}$ is the volume of the extracellular compartment where potassium ions exit to.

Based on our previous example, we can substitute the parameters $N_{AP}=1.37\times 10^{6}$ potassium ions (corresponding to $314\;\mu m^{2}$), $V_{E}=52.6\;\mu m^{3}$, and $N_{A}=6.02\times 10^{23}$ mol^−1^ into Equation (14) to get ${\left[K\right]}_{AP}=4.3\times 10^{-2}$ mmol/L. We give this example to make it easier to follow the subsequent ideas to facilitate an understanding the concept of a quantum synapse between neurons.

When potassium ions exit to the extracellular compartment, the average number of potassium ions $N_{K}$ that can hit a single closed channel in the membrane of an adjacent unstimulated neuron can be calculated by the following equation:

(15) $$N_{K}=\frac{N_{AP}}{D},$$

where *D* is the channel density and $N_{AP}$ is the number of potassium ions that exit through a specific surface area of the neuronal membrane. When Equation (15) is applied, it is important to make sure that the surface area unit in the quantities of $N_{AP}$ and *D* is the same. According to our previous example, when $N_{AP}=4.36\times 10^{3}$ ions/$\mu m^{2}$ and $D=10^{2}$ channels/$\mu m^{2}$ [44,45] (which corresponds to $D=10^{10}$ channels/cm^2^), $N_{K}=44$ ions, which is the number of potassium ions that hit a single closed channel. Thus, the number of potassium ions $N_{K}$ corresponds to the change in the extracellular potassium concentration of $4.3\times 10^{-2}$ mmol/L. As the change in the extracellular potassium concentration increases, the average number of potassium ions hitting the channel increases.

If this minute concentration is substituted in Equation (11), there will be almost no effect on the membrane potential of inner hair cells. However, we will show that the concept of the quantum synapse allows for this small change in potassium concentration to depolarize the membrane and induce an action potential. This is a unique feature of the quantum synapse that makes it distinct from classical electrical communication between neurons.

Next, we will calculate the threshold value of quantum tunneling $T_{Q(Thr)}$ that yields a threshold value of quantum conductance that can depolarize the membrane to the threshold value of potential $V_{m(Thr)}$, inducing an action potential. The $V_{m(Thr)}$ will be assumed to be 55 mV.

The following equation can be used to obtain a relationship between $T_{Q(Thr)}$ and ${\left[K\right]}_{AP}$:

(16) $$MC_{Na}{\left[Na\right]}_{E}+MC_{K}{\left[K\right]}_{E}+{\left[K\right]}_{AP}MC_{Q-K(E)}=e^{\frac{-FV_{m(Thr)}}{RT}}(MC_{Na}{\left[Na\right]}_{I}+MC_{K}{\left[K\right]}_{I}),$$

For the sake of simplicity, we will assume that one channel of the total channels in 1 $\mu m^{2}$ is enough to depolarize the membrane potential to the threshold value. Therefore, $D=10^{8}$ channels/cm^2^ (which corresponds to 1 channel/$\mu m^{2}$) will be substituted in Equation (16). Accordingly, by substituting the values of concentrations and conductance in Equation (16), the relationship between $T_{Q(Thr)}$ and ${\left[K\right]}_{AP}$ can be obtained:

(17) $$T_{Q(Thr)}=\frac{9.64\times 10^{-7}}{{\left[K\right]}_{AP}},$$

If ${\left[K\right]}_{AP}=4.3\times 10^{-2}$ mmol/L is substituted into Equation (17), then the threshold value of quantum tunneling $T_{Q(Thr)}=2.24\times 10^{-5}$. This means that if at least one channel in a surface area of 1 $\mu m^{2}$ is required to induce an action potential, then at least a fraction of $2.24\times 10^{-5}$ from the total potassium ions hitting the channel must tunnel through the closed gate to depolarize the membrane potential sufficiently to induce an action potential. As was explained before, this change in membrane potential corresponds to around 44 potassium ions, which hit a single closed channel. Then, if at least one potassium *ion* from the total 44 potassium ions tunnels through the closed gate, then the minimum tunneling fraction will be $\frac{1}{44}=2.27\times 10^{-2}$. If this minimum fraction is compared with $T_{Q(Thr)}=2.24\times 10^{-5}$, which represents the minimum tunneling fraction required to induce an action potential from at least one channel in 1 $\mu m^{2}$, it is clear that the process of tunneling can induce an action potential since $2.27\times 10^{-2}>2.24\times 10^{-5}$. Therefore, we aim to calculate the probability of achieving this significant fraction of tunneling based on the actual tunneling probability, as shown in Equation (4). Since the action potential is transmitted through a neuron, there will be many chances available for potassium ions to tunnel through the closed channels in the membrane of unstimulated demyelinated neurons. This implies that along the surface area available for potassium *ion* tunneling, there will be a probability that at least one potassium *ion* from the total number hitting a channel (e.g., 44 ions here) will succeed to tunnel and induce an action potential. In this case, the tunneling fraction will be $2.27\times 10^{-2}$(1/44), which is higher than the threshold value of quantum tunneling, $2.24\times 10^{-5}$.

To calculate the probability of the induction of an action potential in an adjacent unstimulated neuron by another neuron carrying the signal of the action potential (AP), the Bernoulli trials equation can be used as follows:

(18) $$P(Z)=\frac{N!P^{Z}{\left(1-P\right)}^{N-Z}}{(N-Z)!Z!},$$

where *Z* is the number of trials that must be met or obtained, *N* is the total number of available trials, *P* is the probability of obtaining a successful trial, and *P(Z)* is the probability of obtaining *Z* number of successful trials. When *Z* = 0, then:

(19) $$P(0)={\left(1-P\right)}^{N},$$

Accordingly, when an action potential is transmitted through a neuron and an increase in the extracellular potassium ions occurs, then the probability that at least one potassium *ion* from the total number $N_{K}$ hitting a single closed channel will succeed and tunnel through the closed gate is:

(20) $$P_{1}=1-{\left(1-T_{Q}\right)}^{N_{K}},$$

where $P_{1}$ is the probability of inducing an action potential by one channel via quantum tunneling of at least one potassium *ion* and $T_{Q}$ is the quantum tunneling probability of potassium ions indicated in Equation (7).

Then, the probability that at least one closed channel from the total number of channels $D_{\mu m^{2}}$ in 1 $\mu m^{2}$ is tunneled by at least one potassium *ion* is:

(21) $$P_{2}=1-{\left(1-P_{1}\right)}^{D_{\mu m^{2}}},$$

where $P_{2}$ is the probability of inducing an action potential in a surface area of 1 $\mu m^{2}$ via quantum tunneling of at least one potassium *ion* through at least one closed channel.

Eventually, the probability of inducing an action potential in at least one area of 1 $\mu m^{2}$ from the total number of surface areas $N_{\mu m^{2}}$ is:

(22) $$P_{3}=1-{\left(1-P_{2}\right)}^{N_{\mu m^{2}}},$$

where $P_{3}$ is the probability of inducing an action potential in 1 $\mu m^{2}$ from the total number of surface areas $N_{\mu m^{2}}$. $P_{3}$ represents the eventual probability of action potential induction along the surface area available for the quantum tunneling of potassium ions.

The total number of surface areas in 1 $\mu m^{2}$ can be calculated by the following equation:

(23) $$N_{\mu m^{2}}=\frac{A}{1\;\mu m^{2}},$$

where A is the surface area (in $\mu m^{2}$) that has been demyelinated and is available for quantum tunneling through its exposed potassium channels. For example, if $A=10^{-10}$ m^2^ = 100 $\mu m^{2}$, then $N_{\mu m^{2}}=100$. This means that there are 100 areas available for the quantum tunneling of potassium ions to induce an action potential. Inducing an action potential in at least one area will be enough to transmit the action potential to the next areas on the same neuron until it reaches the brain hearing centers.

In the results section, we will set different values for the variables that determine the probability of inducing an action potential, which are described in the previous equations. This will help us to provide a more comprehensive understanding of the influence of these variables on the probability of action potential (AP) induction.

## 3. Results

### 3.1. Quantum Tunneling-Induced Membrane Depolarization

Equations (12) and (13) can be used to evaluate the influence of the drop in the barrier height *G* of the closed gate on the membrane potential of the inner hair cell. By substituting the values of concentration and leak conductance and substituting the equation of quantum conductance into Equation (12), the quantum version of the GHK equation for potassium and sodium *ion* is as follows:

(24) $$3.9+{\left[ion\right]}_{E}3.88\times 10^{-2}\times D\times e^{\frac{-\sqrt{8\pi^{2}m}}{\hbar }L(\sqrt{G}-\sqrt{q\frac{V_{m}}{n}+\frac{3}{2}K_{B}T})}=e^{-37.45V_{m}}(60.15+{\left[ion\right]}_{I}3.88\times 10^{-2}\times D\times e^{\frac{-\sqrt{8\pi^{2}m}}{\hbar }L(\sqrt{G}-\sqrt{\frac{3}{2}K_{B}T})})$$

Regarding the quantum version of this equation for calcium ions, as shown in Equation (13), we will consider H2=0 because the concentration of intracellular calcium ions is much lower than that of other ions. Additionally, the quantum tunneling probability of intracellular ions is much lower than the quantum tunneling probability of extracellular ions according to Equations (7) and (8). This is because extracellular ions have higher kinetic energy than the intracellular ions; this is especially true for calcium ions since they are divalent ions. This can be easily checked by substituting the same values to calculate the tunneling probability for extracellular and intracellular calcium ions using Equations (7) and (8).

We make this modification to reduce the mathematical complexity of the equation while maintaining the reasonability and consistency of the results.

Therefore, the quantum version of the GHK equation for calcium ions can be written as the following:

(25) $$-56.25+\sqrt{4.1\times 10^{3}+52.3\times D\times e^{\frac{-\sqrt{8\pi^{2}m}}{\hbar }L(\sqrt{G}-\sqrt{q_{Ca}\frac{V_{m}}{n}+\frac{3}{2}K_{B}T})}}=120.3e^{-37.45V_{m}},$$

where ${\left[Ca\right]}_{E}=1.4$ mmol/L [44,45] and $q_{Ca}=3.2\times 10^{-19}$ C.

According to experimental results concerning the barrier height of the closed gate while the ions are passing through [37,38,39], it is clear that there is no definitive value of *G* because the barrier height depends on the hydrophobicity of the pore residues and the pore radius. However, these results indicate that the barrier height is within the order of magnitude of $10^{-20}$ J (Kcal/mol = $0.69\times 10^{-20}$ J or KJ/mol = $0.17\times 10^{-20}$ J) [30,31,37,38]. Therefore, we will use the range $(1-4)\times 10^{-20}$ J to investigate the effect of the drop in barrier height *G* on the membrane potential under the influence of the quantum tunneling of ions. The risk factors of tinnitus, including noise trauma, aging, ischemia, and inflammation, can affect the integrity of the cellular membrane and make the voltage-gated channels leaky, which is reflected by a drop in the barrier energy of the closed gate [14,54,55,56,57]. Therefore, we aim to show how the drop in the barrier height, which is mediated by the risk factors of tinnitus, can depolarize the membrane potential of inner hair cells.

Furthermore, the length of the gate *L* at which *U*(*L*) = 0.42 *G* depends on the number of amino acids that form the hydrophobic pore. From the experimentally obtained plots, the length of the gate can be estimated as $5\times 10^{-10}$ m for the three residues that form the hydrophobic gate [37,38]. Hence, for a gate with one residue [23,24,25], the length of the gate *L* can be estimated to be around $1.5\times 10^{-10}$ m. Moreover, the experimentally obtained curve for the energy barrier of the gate is not a perfect symmetric Eckart potential [30,31,32,33,37,38,39]. Thus, the symmetric Eckart potential used in the present study may underestimate the tunneling probability and the quantum conductance. Accordingly, to account for the short length of the gate in the voltage-gated channels and the asymmetry in the experimental curves, we will use the range of $(0-2)\times 10^{-10}$ m with an average value of $1\times 10^{-10}$ m and four setting values ($L=0.5\times 10^{-10}$ m, $L=1\times 10^{-10}$ m, $L=1.5\times 10^{-10}$ m, and $L=2\times 10^{-10}$ m) for the purpose of this investigation. Moreover, when the quantum tunneling-induced membrane depolarization is investigated, $D=10^{10}$ channels/cm^2^ [44,45], which corresponds to $D=10^{2}$ channels/$\mu m^{2}$, will be substituted.

#### 3.1.1. The Influence of the Length of the Gate on Quantum Tunneling-Induced Membrane Depolarization

**Figure 5:**
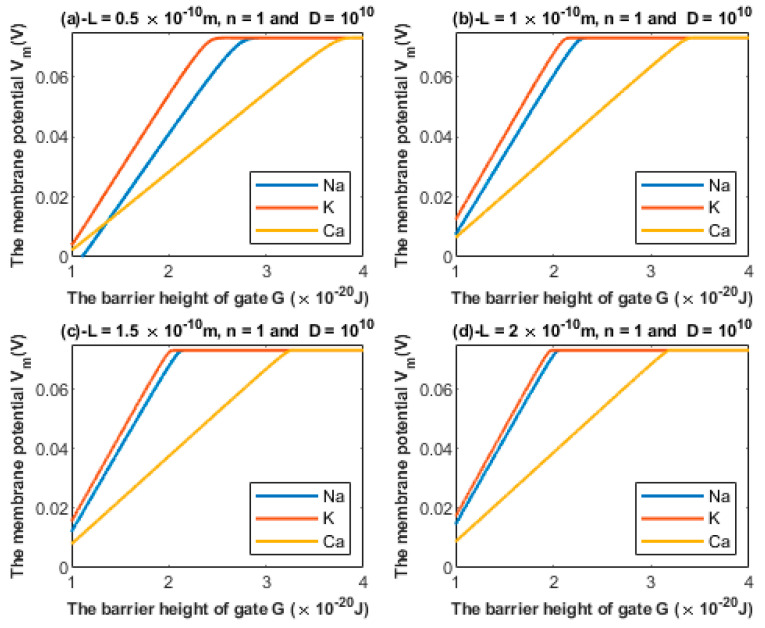
Relationship between the barrier height of the gate *G* and the membrane potential according to the values specified above the figure and varying values of gate length: (**a**) $L=0.5\times 10^{-10}$ m, (**b**) $L=1\times 10^{-10}$ m, (**c**) $L=1.5\times 10^{-10}$ m, and (**d**) $L=2\times 10^{-10}$ m. The figure clearly delineates the ability of all the three cations to depolarize the membrane potential.

According to Equations (24) and (25), the relationship between the barrier height *G* and the membrane potential of an inner hair cell can be investigated at different values of gate length *L* (Figure 5).

#### 3.1.2. The Influence of Gate Location on Quantum Tunneling-Induced Membrane Depolarization

**Figure 6:**
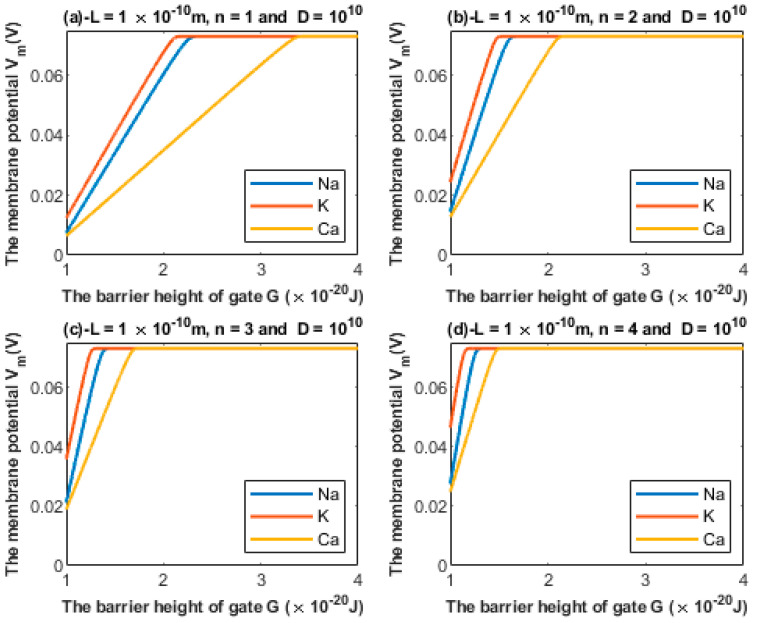
Relationship between the barrier height of the gate *G* and the membrane potential according to the values specified above the figure and varying values of gate location: (**a**) *n* = 1, (**b**) *n* = 2, (**c**) *n* = 3, and (**d**) *n* = 4. The figure clearly delineates the ability of all the three cations to depolarize the membrane potential.

According to Equations (24) and (25), the relationship between the barrier height *G* and the membrane potential of an inner hair cell can be investigated at different values of gate location *n* (Figure 6).

### 3.2. The Probability of Action Potential Induction via Quantum Tunneling of Potassium Ions (the Formation of a Quantum Synapse)

By utilizing Equations (20)–(22), the probability of action potential (AP) induction, which is represented by $P_{3}$ in Equation (22), can be investigated under the influence of different factors. We will evaluate the probability of action potential induction by using the following setting values: $L=1\times 10^{-10}$ m, $V_{m}=0.07$ V, $N_{K}=100$, D=100 channels/$\mu m^{2}$, and $N_{\mu m^{2}}=100$. When we use these setting values, we will choose one of these variables to set at different values in order to investigate the probability of AP induction. See the following figures.

**Figure 7:**
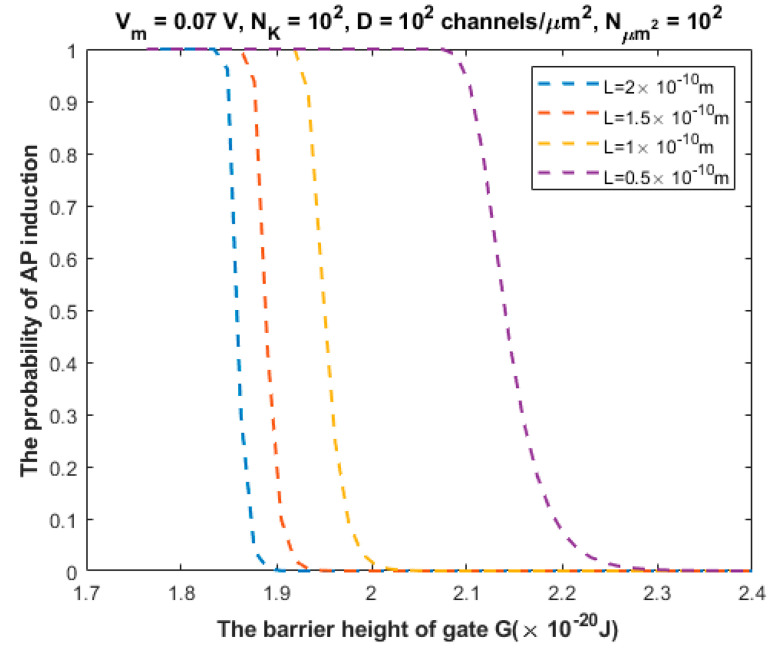
Relationship between the barrier height of the gate *G* and the probability of action potential (AP) induction according to the values specified above the figure and varying values of gate length L.

The relationship between the probability of action potential induction and the barrier height of the gate *G* can be evaluated at different values of gate length *L* (Figure 7).

**Figure 8:**
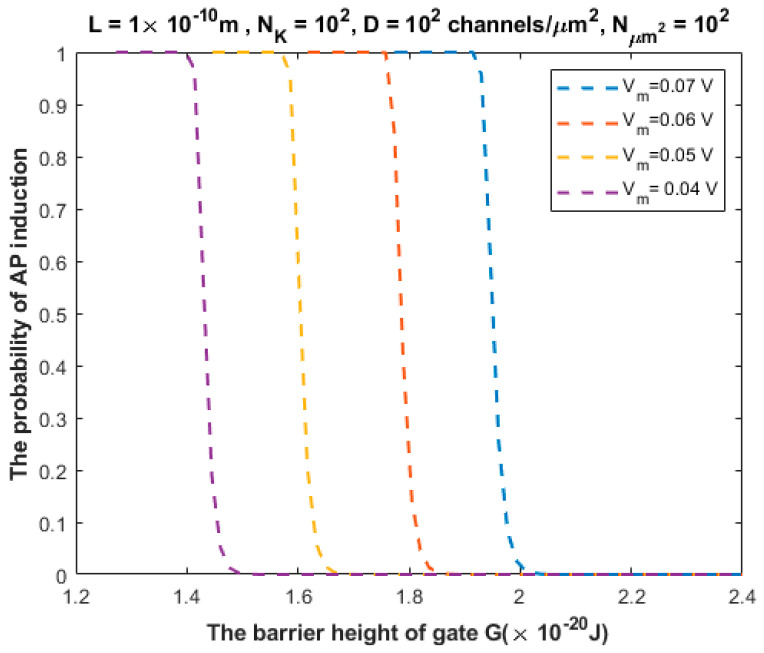
Relationship between the barrier height of the gate *G* and the probability of action potential (AP) induction according to the values specified above the figure and varying values of membrane potential.

The relationship between the probability of action potential induction and the barrier height of the gate *G* can be evaluated at different values of membrane potential (Figure 8).

**Figure 9:**
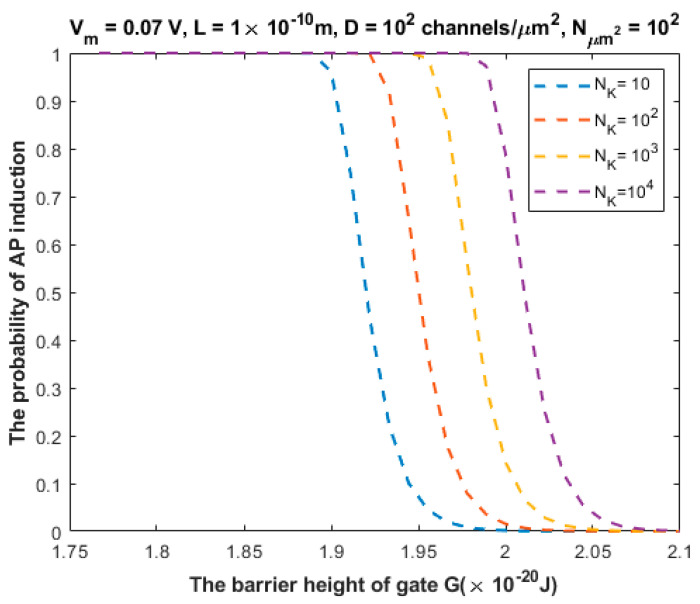
Relationship between the barrier height of the gate *G* and the probability of action potential (AP) induction according to the values specified above the figure and varying values of the number of potassium ions $N_{K}$.

The relationship between the probability of action potential induction and the barrier height of the gate *G* can be evaluated at different values of $N_{K}$ (Figure 9).

**Figure 10:**
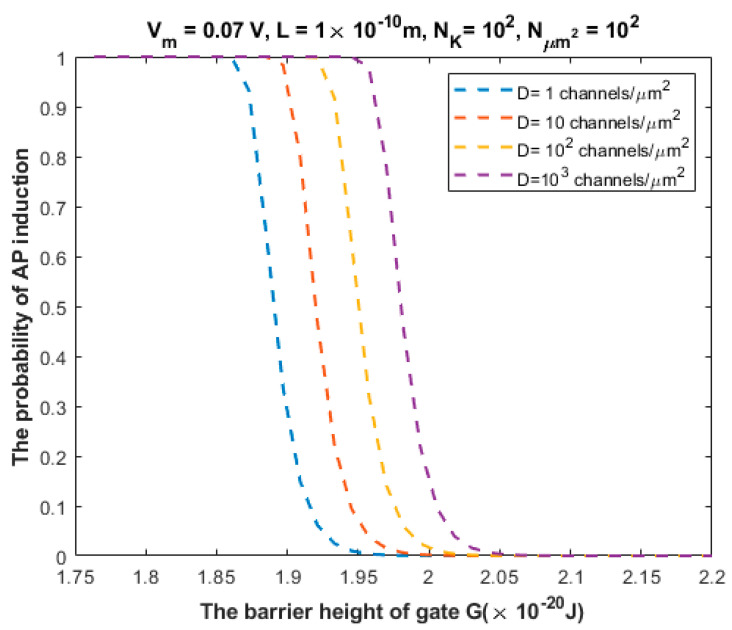
Relationship between the barrier height of the gate *G* and the probability of action potential (AP) induction according to the values specified above the figure and varying values of channel density *D*.

The relationship between the probability of action potential induction and the barrier height of the gate *G* can be evaluated at different values of $D_{\mu m^{2}}$ (Figure 10).

**Figure 11:**
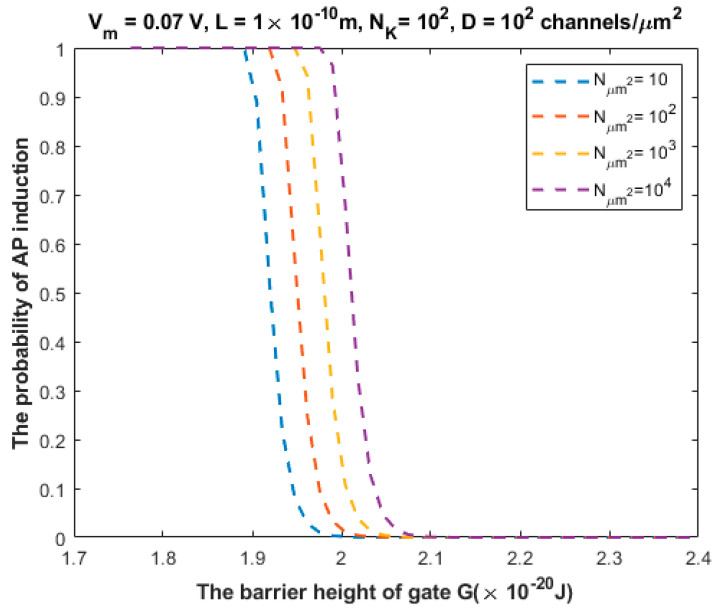
Relationship between the barrier height of the gate *G* and the probability of action potential (AP) induction according to the values specified above the figure and varying values of $N_{\mu m^{2}}$.

The relationship between the probability of action potential induction and the barrier height of the gate *G* can be evaluated at different values of $N_{\mu m^{2}}$ (Figure 11).

## 4. Discussion

The present study proposed a quantum investigational approach to understand the pathophysiology of tinnitus and provide a more comprehensive view on its pathogenesis and clinical manifestations. We focused on the voltage-gated channel as a molecular target to which the quantum model was applied.

The quantum model utilized in this study is the quantum tunneling of ions through closed voltage-gated channels. This model states that ions have a non-zero probability of passing through structurally closed channels [13,14,15]. The quantum tunneling of ions results in quantum currents and the quantum conductance of the corresponding channels. Moreover, it was found that these ions were not able to affect the membrane potential of excitable tissue at normal physiological parameters, at which the barrier height of the gate is large enough to block both the classical permeation and the quantum tunneling of ions, especially for sodium, potassium, and calcium ions due to their large masses [13,14,15]. However, under certain pathological events, the barrier height of the closed gate drops critically, amplifying the quantum tunneling, which results in a significant quantum conductance that can depolarize the membrane potential [13,14,15,16,17,18,19]. These pathological events include channelopathies; hypoxia; ischemia; inflammation; mechanical damage, including trauma and stretch; or any factor that can affect the integrity of the cellular membrane and *ion* channel proteins [54,55,56,57].

Interestingly, all the risk factors of tinnitus, including noise trauma, aging, hypertension, vascular disease, autoimmune diseases, and neurodegeneration, fall within the category of the pathological events that decrease the barrier height of the closed gate [1,12]. Hence, the risk factors of tinnitus offer a suitable pathological environment to augment the quantum tunneling of ions, which leads to a quantum conductance that can depolarize the membrane potential. In the results section, we investigated the quantum tunneling of three ions—sodium, potassium, and calcium—and their influence on the resting membrane potential of inner hair cells at the basolateral membrane.

**Figure 12:**
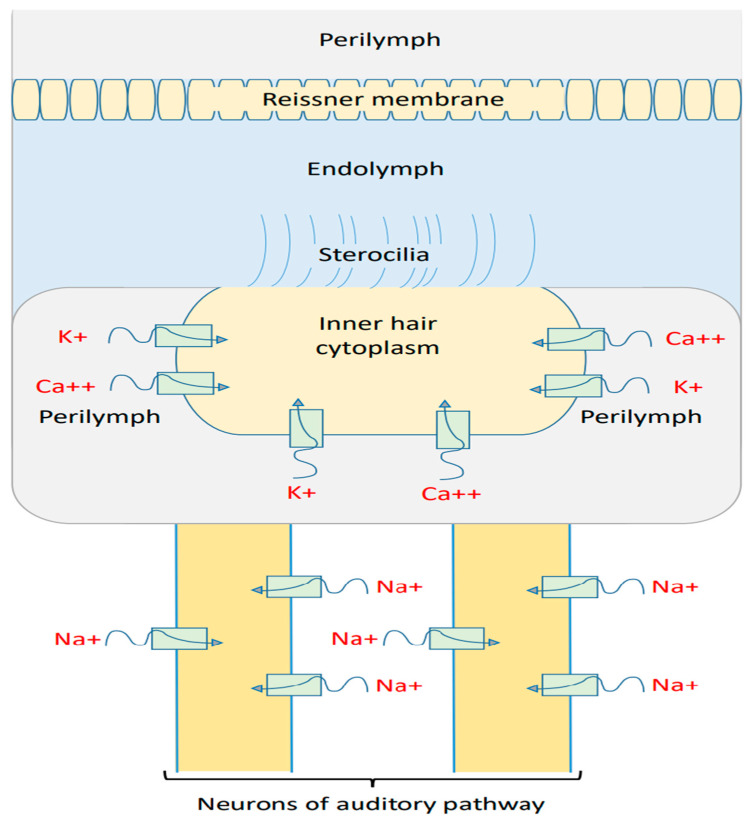
Schematic diagram of the quantum tunneling action of calcium ions, potassium ions, and sodium ions at the basolateral membrane of inner hair cells and through the membrane of auditory pathway neurons. This quantum tunneling of ions can depolarize the membrane potential when the quantum version of the GHK equation is applied.

We previously mentioned the challenges behind providing a consistent mechanism of the membrane depolarization of inner hair cells. These include: (1) The closure of MET channels at the stereocilial membrane results in hyperpolarization instead of depolarization. (2) The discordance between outer hair cells and inner hair cells does not take into consideration the direction of the impingement force of the tectorial membrane on the stereocilia of inner hair cells nor the chronic phases of cellular damage in which harmful effects are involved for both the outer hair cells and inner hair cells. (3) The direct mechanisms affecting the inner hair cells and their membrane potential are not well understood. Therefore, the quantum tunneling model can provide a reasonable mechanism for the depolarization of inner hair cells at the basolateral membrane. Based on Figure 5 and Figure 6, it is obvious that all three ions are able to depolarize the resting membrane potential of IHCs via quantum tunneling and quantum conductance. Moreover, these ions are also able to depolarize the resting membrane potential of the auditory neurons, starting from the cochlea and reaching the brain centers (Figure 12).

Quantum tunneling-induced membrane depolarization is caused by a discrepancy between the extracellular and intracellular ions in terms of tunneling probability, according to Equations (5)–(8). The extracellular ions have higher kinetic energy, and thus a higher tunneling probability and higher quantum conductance. As a consequence, a net influx of cations to the inside of the inner hair cells and neurons occurs. This net influx is expected to depolarize the membrane potential, which is mathematically shown by using the quantum version of the GHK equation.

Additionally, based on Figure 5 and Figure 6, the degree of depolarization varies according to the gate length and gate location. As the gate length and the gate location (*n*) increase, the degree of depolarization induced by all ions decreases. Moreover, it is noticeable that the membrane potential of inner hair cells remains unaffected across a certain range of the barrier height of the gate G until reaching a critical value at which depolarization begins. Furthermore, the three ions vary according to the value at which depolarization begins. Calcium ions can depolarize the membrane potential at higher values of gate energy *G* when compared with sodium and potassium ions; this is attributed to the divalence of calcium ions (+2), which allows them to acquire higher kinetic energy while passing across the membrane potential. In addition, as the energy of the gate *G* decreases, more depolarization is induced in the membrane of inner hair cells.

As the membrane depolarization of IHCs is a main trigger of the spontaneous cochlear activity that contributes to the sensation of tinnitus, the quantum model predicts that the risk factors can also depolarize the membrane potential of the neurons in the auditory pathway. This includes the peripheral and central neurons. Therefore, the quantum model speculates that membrane depolarization increases the electrical signals reaching the central circuits not only through the cochlea, but also the peripheral neurons and even the central neurons.

Thus, quantum tunneling-induced membrane depolarization provides a direct mechanism that explains the depolarization of inner hair cells themselves. Hence, it explains the generation of tinnitus in the chronic phases in which the discordance between inner hair cells and outer hair cells is lost. Additionally, the quantum model involves the peripheral and central neurons in the pathological mechanism of depolarization.

In addition to increasing the electrical activity of the cochlea and auditory neurons, quantum tunneling-induced membrane depolarization can inhibit electrical signaling, particularly with large membrane depolarizations, as shown in Figure 5 and Figure 6. The large membrane depolarization in the inner hair cells increases the number of inactivated calcium channels [58]. As the number of inactivated calcium channels increases, fewer calcium ions enter the inner hair cells in response to the movement of stereocilia. Thus, the release of chemical neurotransmitters that transmit the electrical signals of hearing is reduced. In other words, quantum tunneling through closed channels allows for a persistent calcium influx instead of an oscillatory calcium influx, which is controlled by the classical opening and closing of calcium channels according to the changes in cochlear potential and potassium influx. Furthermore, a large depolarization can decrease the driving force necessary for potassium ions to flow from the endolymph to the inside of the inner hair cells to initiate the transmission of hearing signals. Therefore, the suppressing effects of quantum tunneling-induced membrane depolarization on cochlear activity can explain the hearing loss associated with tinnitus. Another pathological aspect is that the persistent calcium influx into the cytoplasm of inner hair cells, mediated by quantum tunneling, may result in calcium overload, which has harmful effects on inner hair cells, including cell death [59,60]. Calcium-induced cell death can further exacerbate the quantum tunneling-induced membrane depolarization due to a further drop in the barrier height of the gate since the cascades of cell death fall within the pathological categories that affect the integrity of the cellular membrane and render the voltage-gated channels leaky [54,55,56,57].

The second major aspect in the pathophysiology of tinnitus according to our model is the quantum crosstalk between auditory neurons upon demyelination due to noise trauma, presbycusis [12,21], and the other risk factors of tinnitus, including ischemic diseases [61,62], diabetes [63], and neurodegeneration [64]. Once these neurons are demyelinated, the voltage-gated potassium channels become exposed after being covered by the myelin sheath [48,49,50,51]. These exposed potassium channels will be tunneled by the potassium ions that exit into the extracellular fluid from adjacent stimulated neurons. The quantum tunneling of these potassium ions through the exposed channels in the demyelinated neurons can depolarize the membrane potential to the threshold required to induce an action potential. This means that the action potential generated in one neuron can be induced in an unstimulated neuron via the quantum tunneling of potassium ions through the exposed potassium channels.

This quantum crosstalk between demyelinated neurons can be viewed as a “quantum synapse” or quantum signal between them. These signals can elicit an action potential in adjacent unstimulated neurons. As a result, the “quantum synapses” generate a state of hyper-excitability in the neurons of the auditory pathway, including the peripheral and central circuits. This results in a higher rate of electrical signals reaching the brain’s hearing centers, which further contributes to the pathogenesis of tinnitus [1,12]. Moreover, these quantum synapses weaken the spatiotemporal fidelity of the hearing electrical signals transmitted from the cochlea, because the number of stimulated neurons that reach the central circuits and the frequency of action potentials will be higher in this case. The lost spatiotemporal fidelity affects the quality of hearing since the precise fidelity of action potentials is a requirement for hearing to be intact [7]. Accordingly, our model argues that tinnitus is a poor quality of coding the sound signals in the brain centers due to hyper-excitability and the loss of spatiotemporal fidelity.

In fact, the crosstalk between demyelinated auditory neurons has been proposed before [12] as a mechanism for generating tinnitus. This type of crosstalk is referred to as ephaptic coupling or ephaptic interaction [12]. However, the underlying mechanism that explains the formation of such crosstalk is not well-defined [65]. Therefore, in our study, we proposed a well-defined process that underlies the formation of the ‘quantum synapse’, which can be referred to as the ephaptic interaction between neurons.

We investigated the probability of inducing an action potential in an unexcited neuron when an adjacent excited neuron transmits an action potential (Figure 7, Figure 8, Figure 9, Figure 10 and Figure 11). Based on these figures, as the barrier height of the gate decreases, the probability of inducing an action potential in an unstimulated neuron increases, and thus the appearance of quantum synapses is amplified. In addition, many factors modulate the probability of action potential induction, which in turn modulates the severity or the progression of tinnitus [66]. These factors include the gate length, the membrane potential, the number of potassium ions hitting a single channel (which depends on the change in the extracellular potassium concentration during an action potential), the density of potassium channels, and the surface area of demyelination that exposes closed potassium channels to quantum tunneling. In addition, a unique feature of the quantum synapse is that an action potential can be induced by minute changes in extracellular potassium concentrations at the resting state. Indeed, the same minute changes induced at the resting state cannot significantly affect the membrane potential of inner hair cells if the classical version of the GHK equation is applied.

Quantum tunneling-induced membrane depolarization and the formation of quantum synapses can occur at any neuronal level throughout the auditory pathway, beginning from the cochlea and reaching the brain centers [1,12].

As the quantum tunneling model implies the ability of ions to pass through closed gates, it is significant to mention that it has been observed that ions can pass through a structurally closed gate [67,68]. These observations could serve to support the existence of the quantum tunneling of ions because they fit the quantum tunneling model very well, unlike the classical model of *ion* channels, which requires a structurally open channel to permeate ions. However, these results were not interpreted in the context of quantum tunneling because the quantum model was not available at that time. Additionally, the quantum synapse implies a neuronal communication without a chemical or electrical synapse. Interestingly, it seems that ephaptic coupling operates in the same way [69]. Although ephaptic coupling has been observed experimentally, its underlying mechanism seems elusive and is not well defined because the electrical field changes during neuronal firing are not enough to affect the membrane potential of adjacent neurons, and the concentration changes in potassium ions must be high enough to depolarize the membrane potential [65]. According to the similarity between them, we propose that the underlying mechanism behind ephaptic coupling is the formation of the quantum synapse, which guarantees neuronal communication without the requirement of large changes in the endogenous electric field or large changes in the concentration of potassium ions.

Therefore, similar actions to quantum tunneling and the quantum synapse have been observed experimentally; however, as mentioned previously, no concrete mechanisms are available to explain them. Hence, we suggest that these observed actions could be explained in the context of the quantum tunneling model. Therefore, we encourage researchers in the related disciplines to utilize the tunneling model to explore the conductance of channels, especially when they are closed, and to explain certain physiological and pathological entities, particularly if they are not fully understood by the classical electrophysiological concepts, such as tinnitus and other previously investigated physiological, pathological, and pharmacological processes and actions [16,17,18,19].

## 5. Conclusions

**Figure 13:**
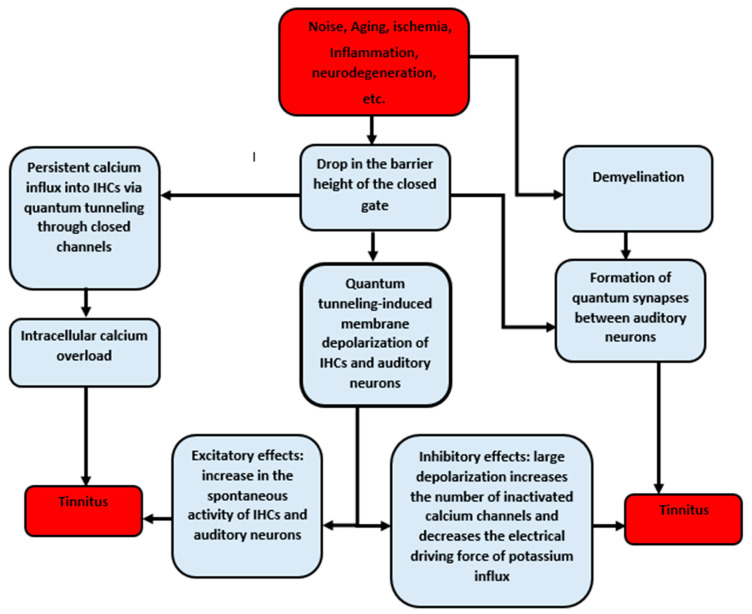
Schematic diagram of the pathophysiology of tinnitus according to the quantum tunneling model of ions.

The quantum tunneling model provides a consistent approach to explain the pathogenesis of tinnitus. The risk factors of tinnitus decrease the barrier height of the closed gate and promote demyelination. As a result, the drop in the energy barrier of the gate augments the quantum tunneling of calcium, potassium, and sodium ions through the closed voltage-gated channels. This leads to the quantum tunneling-induced membrane depolarization of inner hair cells and the auditory neurons and increases the spontaneous activity of the cochlea and neurons. However, the inhibitory effects of this depolarization can also be observed. If a large depolarization takes place, which is what we predict according to the quantum model, it is expected that the flow of potassium ions through the stereocilial membrane will decrease, the number of inactivated calcium channels will increase, and intracellular calcium overload will occur due to the persistent influx of calcium ions to the inner hair cells via quantum tunneling. All these effects suppress the spontaneous activity of the cochlea, which explains the hearing loss associated with tinnitus. On the other hand, demyelination and the drop of the gate energy aid in the formation of quantum synapses between the neurons of the auditory pathway. The formation of quantum synapses will impair the spatiotemporal fidelity of the sound signals that are transmitted to the central auditory circuits, resulting in the poor coding of sound signals, which is interpreted by the brain as tinnitus (Figure 13).

## Data Availability

Not applicable.
